# Gut microbiota influence in type 2 diabetes mellitus (T2DM)

**DOI:** 10.1186/s13099-021-00446-0

**Published:** 2021-08-06

**Authors:** A. L. Cunningham, J. W. Stephens, D. A. Harris

**Affiliations:** 1 Department of Surgery, Swansea Bay University Health Board, Singleton Hospital, Swansea, SA2 8QA Wales; 2 School of Medicine, Swansea University Medical School, Institute of Life Science 2, Swansea, SA2 8QA Wales

**Keywords:** Gut microbiota, Type two diabetes mellitus

## Abstract

A strong and expanding evidence base supports the influence of gut microbiota in human metabolism. Altered glucose homeostasis is associated with altered gut microbiota, and is clearly associated with the development of type 2 diabetes mellitus (T2DM) and associated complications. Understanding the causal association between gut microbiota and metabolic risk has the potential role of identifying susceptible individuals to allow early targeted intervention.

## Background

The global prevalence of type 2 diabetes mellitus (T2DM) is projected to grow beyond 700 million patients by 2045 [1], at a cost to society of greater than two trillion US dollars [2]. The World Health Organisation (WHO) and the United Nations (UN) have made T2DM prevention a top health priority [3, 4]. One in fifteen individuals in the United Kingdom (UK) has a diagnosis of diabetes, of which T2DM accounts for around 90%, a current cohort of greater than three million people. Population estimations suggest one million patients already suffer from undiagnosed T2DM. Of these cases, 60% are considered preventable through lifestyle and dietary intervention [5]. Obesity, central adiposity and body mass index (BMI) play a pivotal role in the pathophysiology of T2DM, a chronic metabolic disease characterised by hyperglycaemia and associated with insulin resistance and/or insufficient pancreatic insulin production [6]. Unrecognised or suboptimal T2DM may lead to both micro- and macrovascular complications associated with hypertension, renal failure, susceptibility to infection, limb amputations and blindness with their subsequent disability.

## Microbiota

In 2001, Joshua Lederberg, a former Nobel Prize winner, first defined the ‘human microbiome’ as an ecological community of commensal, symbiotic and pathogenic microorganisms that collectively share our body space [7]. Human health is strongly influenced by microbiota that are co-habiting with our body [8]. An adult human is colonised by approximately 100 trillion microbes found predominantly in the gastrointestinal tract (GIT), of which the largest population resides in the colon.

The majority of bacterial species cannot be cultured, however, the advancement of microbial analysis techniques and the use of rodent models has enabled the investigation of the role of gut microbiota in the pathogenesis of T2DM. Although rodents and humans differ in certain aspects of their physiology, animal models provide valuable opportunities to conduct investigations that cannot be undertaken in humans [9]. The vast majority of gut microbiota belong to four main families (phyla):—Firmicutes, Bacteroidetes, Proteobacteria and Actinobacteria [10–12]. Other smaller but relevant phyla include the Verrucomicrobia and Fusobacteria [13]. Under normal physiological conditions, Firmicutes make up the greatest proportion of the gut microbiota (64%), followed by the Bacteroidetes (23%), Proteobacteria (8%) and lastly Actinobacteria (3%). Evidence suggests that gut microbiota can influence human health either directly or indirectly [14, 15] and that disruption to stable communities may increase the prevalence of pro-inflammatory conditions such as obesity, inflammatory bowel disease, T2DM, arthritis and cancer [16]. Both animal models and humans with T2DM have demonstrated compositional changes within their microbiota profiles, which is most apparent at phylum and class levels [17, 18]. Confounding factors such as geographical location, culture, diet, health status and medication-use however have led to difficulty in identifying a ‘common’ microbiota profile associated with T2DM [19]. Complete bacterial counts and gene numbers are similar between T2DM patients and healthy controls [17, 20], but this diversity significantly declines in T2DM [21–23]. It is unlikely that a single microbe species plays a dominant role in determining the risk of T2DM [24].

Groups of subjects with and without T2DM have contrasting microbiota findings in terms of phyla composition (summarised in Table 1). Significantly lower relative abundances of Firmicutes, compared with a much higher proportion of Bacteroidetes and Proteobacteria has been reported in subjects with T2DM [17]. The Bacteroidetes to Firmicutes ratio (B/F ratio) is associated with increased plasma glucose following an oral glucose load [17]. Conversely, other research groups describe the abundance of Firmicutes and Proteobacteria as significantly increased, while Bacteroidetes is greatly reduced resulting in an enhanced F/B ratio in T2DM compared to non-diabetic individuals [23, 25]. Zhao et al., clarified the enhanced F/B ratio in T2DM subjects further, comparing complicated and uncomplicated T2DM cohorts and displaying an increased F/B ratio in favour of the complicated cohort, as expected [25]. Other groups have found no significant differences in microbiota [26]. Opportunistic pathogens are frequently described in T2DM microbiota communities including the species *Bacteroides caccae*, *Clostridium hathewayi*, *Clostridium ramosum*, *Clostridium symbiosum*, *Eggerthella lenta* and *Escherichia coli* [18, 26, 27].

**Table 1:** Microbiota community differences between participant cohorts

Participant cohort	Microbiota differences
Normal physiological conditions [10–12]	64% Firmicutes 23% Bacteroidetes 8% Proteobacteria 3% Actinobacteria
T2DM cohort [17]	↓ Firmicutes ↑ Bacteroidetes ↑ B/F ratio ↑ Proteobacteria
T2DM cohort [23, 25]	↑ Firmicutes ↑ Proteobacteria ↓ Bacteroidetes ↑ F/B ratio
Complicated T2DM vs uncomplicated T2DM [25]	↑ F/B ratio
T2DM cohort [26]	No difference

Specific genera with relatively high abundances in T2DM patients have also been identified. See Table 2 for summary. These include *Blautia*, *Coprococcus*, *Sporobacter*, *Abiotrophia*, *Peptostreptococcus*, *Parasutterella* and *Collinsella* [25, 26, 28].

**Table 2:** Individual microbiota differences in T2DM cohorts

Patient cohort	Microbiota findings
T2DM [18, 26, 27]	↑ opportunistic pathogens Bacteroides caccae, Clostridium hathewayi, Clostridium ramosum, Clostridium symbiosum, Eggerthella lenta, Escherichia coli
T2DM [24, 26, 28]	↑ Blautia, Coprococcus, Sporobacter, Abiotrophia, Peptostreptococcus, Parasutterella, Collinsella
T2DM [18, 21–23, 27, 120]	↓ butyrate-producing microbes Eubacterium rectale, Faecali prausnitzii, Roseburia intestinalis, Roseburia inulinivorans, Ruminococcus and Subdoligranulum
T2DM [23, 25, 34, 35]	↓ Bacteroides, Prevotella, Bifidobacterium
T2DM [20, 23, 35]	↑ Lactobacillus ↓ × 5 clostridium species
T2DM [25]	↓ Akkermansia
Pre-diabetic [43]	↓ Akkermansia, Clostridium ↑ Ruminococcus, Streptococcus

Butyrate producing microbes are particularly depleted in patients diagnosed with T2DM specifically the Clostridiales order, including the genera *Ruminococcus* and *Subdoligranulum*, and the species *Eubacterium rectale*, *Faecali prausnitzii*, *Roseburia intestinalis* and *Roseburia inulinivorans* [18, 21–23, 27]. Butyrate is well understood to benefit host homeostasis and will be discussed in further detail later in the review. The genera *Bacteroides*, *Prevotella* and *Bifidobacterium* are found in significantly less numbers in T2DM patients [23, 25, 33, 34]. The genus *Bifidobacterium* is known to provide significant health benefits including the ability to improve intestinal permeability thereby lowering circulating levels of endotoxin and reducing systemic inflammation. This correlates with the improvement of host glucose tolerance and glucose-induced insulin secretion, and reduces inflammation [35–37].

A female European T2DM cohort displayed much greater numbers of the *Lactobacillus* species and a decline in the abundance of five *Clostridium* species [20]. Similar conclusions were also reported in two other studies [23, 34]. An increase in the population of the genus *Lactobacillus* correlates positively with lower fasting glucose levels and improved glycated haemoglobin (HbA1c) levels. Both species have no relationship with BMI [20]. Supplementing diabetic rodents with strains of the species *Clostridium butyricum* led to an improvement in circulating glucose levels, decreased systemic insulin resistance and inflammation, increased mitochondrial metabolism and a significant reduction in gut disruption [38].

The species *Akkermansia muciniphila* and *Faecali prausnitzii* appear to provide protection against the development of T2DM [27, 39, 40]. The genus *Akkermansia* plays a critical role in maintaining the integrity of the mucin layer and reducing inflammation [41]. Mucins are large, highly glycosylated proteins that partake in luminal protection of the GIT leading to a reduction in bacterial translocation and improving the storage of fat, adipose tissue metabolism and glucose homeostasis [41]. T2DM patients display significantly lower levels of *Akkermansia* [25]. Supplementing rodents with oligo-fructose (resulting in a secondary increase in *Akkermansia*) or direct treatment with *Akkermansia* improves their overall metabolic status [41]. Initiating T2DM treatment also appeared to directly initiate an increase in the abundance of *Faecali prausnitzii*, a secondary reduction in systemic inflammation and an improvement in insulin resistance [27]. Patients with pre-diabetes also demonstrate similar findings in their microbiota communities including a decrease in microbial diversity; depletion in the numbers of the genera *Akkermansia* and *Clostridium*; and increases in *Ruminococcus* and *Streptococcus* [42].

If a ‘common’ microbiota profile can be identified for T2DM, it could be possible to utilise microbial biomarkers alongside clinical parameters in a machine learning prediction model to distinguish patients at risk of T2DM with reliable diagnostic accuracy. Secondly, if this model proves successful, the selected microbial biomarkers could be used to monitor patients’ glycaemic control and the introduction of new therapeutics.

## Type 1 diabetes mellitus

Type 1 diabetes mellitus (T1DM) is a cellular-mediated autoimmune disease in which the destruction of pancreatic β-cells causes insulin deficiency resulting in hyperglycaemia and a potential for ketoacidosis. Autoimmune destruction of β-cells has strong genetic predispositions and are also related to environmental constituents that are still poorly understood [43, 44]. The development of T1DM has been linked to aberrant intestinal microbiota, microbial-induced butyrate production, a disrupted intestinal mucosal barrier, and altered mucosal immunity [45, 46].

Currently there are several large prospective epidemiological studies in T1DM children aiming to identify and investigate environmental causes. The Environmental Determinants of Diabetes in the Young (TEDDY) study is the largest, with the aim of following several thousand newborns with a genetic predisposition for T1DM or a first-degree relative with T1DM [47]. Initial analysis has demonstrated that the presence of five bacterial genera is associated with the early development of T1DM, the genus *Parabacteroides* being the most significant. Secondly, eleven bacterial genera were depleted in the T1DM cohort, including four unclassified Ruminococcaceae, *Lactococcus*, *Streptococcus* and *Akermansia* [48].

In the islet autoimmunity (IA) case–control cohort, healthy controls contained higher levels of the species *Lactobacillus rhamnosus* and *Bifidobacterium dentium*, whereas IA cases had higher abundances of *Streptococcus* *mitis/oralis/pneumoniae* species [47]. A reduction in bacterial pathways for the production of short-chain fatty acids (SCFAs) such as butyrate in children who developed islet autoantibodies or T1DM were observed [47]. The relevance of the SCFAs is discussed in detail later in the review. Modifying the microbiota community is an interesting possibility in order to prevent T1DM development. Results from the TEDDY study demonstrated a decrease in islet autoimmunity in children given probiotics in early infancy [49]. Further studies are ongoing, however there is still a considerable lack of literature positively connecting microbiota dysbiosis as a predictor in the pathogenesis of T1DM [50].

## Medication induced changes in gut microbiota composition

Gut microbial composition is highly variable between individuals and is continuously modified by endogenous and exogenous factors [51]. Geographic and environmental factors such as diet, illness, lifestyle, hygiene and medications can contribute to changes [52–54]. Antibiotic treatments have the ability to disrupt the gut microbiota community for several years after administration [55]. A population-wide case–control study performed in Scandinavia illustrated a strong association between antibiotic exposure and the development of subsequent T2DM. A relationship between T2DM diagnosis and the number of antibiotic prescriptions was also observed [56]. Further detailed work is required to establish association or causation. It is possible that antibiotics may predispose patients to the development of T2DM, however patients at-risk of T2DM may be more susceptible to illness in the years prior to diagnosis [56]. Vrieze et al., studied the effects of antibiotic treatment on the gut microbiota and the resulting effect on metabolic parameters in patients with obesity and insulin resistance. Vancomycin significantly lowered microbial diversity, decreased the abundance of Firmicutes, improved the numbers of Proteobacteria, particularly the genus *Lactobacillus* and decreased peripheral insulin sensitivity [57].

Glucose lowering medication including the biguanides, alpha-glucosidase inhibitors, incretin-based drugs, glucagon-like peptide 1 (GLP-1) receptor agonists, dipeptidyl peptidase-4 inhibitors and thiazolidinediones can all influence the gut microbiota [58]. Metformin is one of the most widely prescribed oral medications for patients with T2DM and does not intentionally modify gut microbiota. However, there is growing evidence to indicate that some effects may be enhanced by the microbiota [59, 60]. Metformin increases the relative abundance of the genera *Akkermansia*, *Bifidobacterium* and *Lactobacillus* [59–61]. Other enriched genre associations include *Bacteroides*, *Butyricoccus*, *Prevotella*, *Megasphaera* and *Butyrivibrio* [60]. These particular microbiota all have the ability to produce SCFAs. Metformin treatment results in improved microbial diversity, rapid changes in gut microbiota composition and improves intestinal function by enhancing SCFA production, promoting the activity of endocrine cells, regulating bile acid (BA) turnover, and reducing endotoxemia [60]. Short-term metformin treatment is associated with significantly lowered abundance of the species *Bacteroides fragilis* resulting in secondary increases of BA glycoursodexoycholic acid (GUDCA) levels in the gut. GUDCA inhibits intestinal farnesoid X receptor (FXR) signalling leading to an improvement in glucose tolerance. Reintroducing *Bacteroides fragilis* reverses the improvements seen in glucose metabolism with metformin usage [62]. Other diabetic medications have not been as widely scrutinised as metformin treatment. Glibenclamide has only minor effects on gut microbiota alpha diversity. It increases the relative abundance of the family *Paraprevotellaceae* and *Prevotella* species [63]. Neither dapagliflozin or gliclazide have been shown to alter gut microbiota in T2DM patients to any significant extent when used in combination with metformin [64]. In high-fat dietary fed (HFD) rodents, liraglutide reduces gut microbial diversity and lowers the abundance of the phyla Bacteroidetes, Proteobacteria and Actinobacteria [65]. Decreases in the relative abundance of all obesity-related phylotypes (the genera *Romboutsia* and *Ruminiclostridium*, and the family *Erysipelotrichaceae*) were also noted, accompanied with an enrichment in the lean-related genre *Blautia* and *Coprococcus* [66]. Patients receiving GLP-1 agonists in combination with metformin have higher abundances of the genus *Akkermansia* than those on single treatment liraglutide [67].

## Gut microbiota impact on glucose and insulin metabolism

**Fig. 1:**
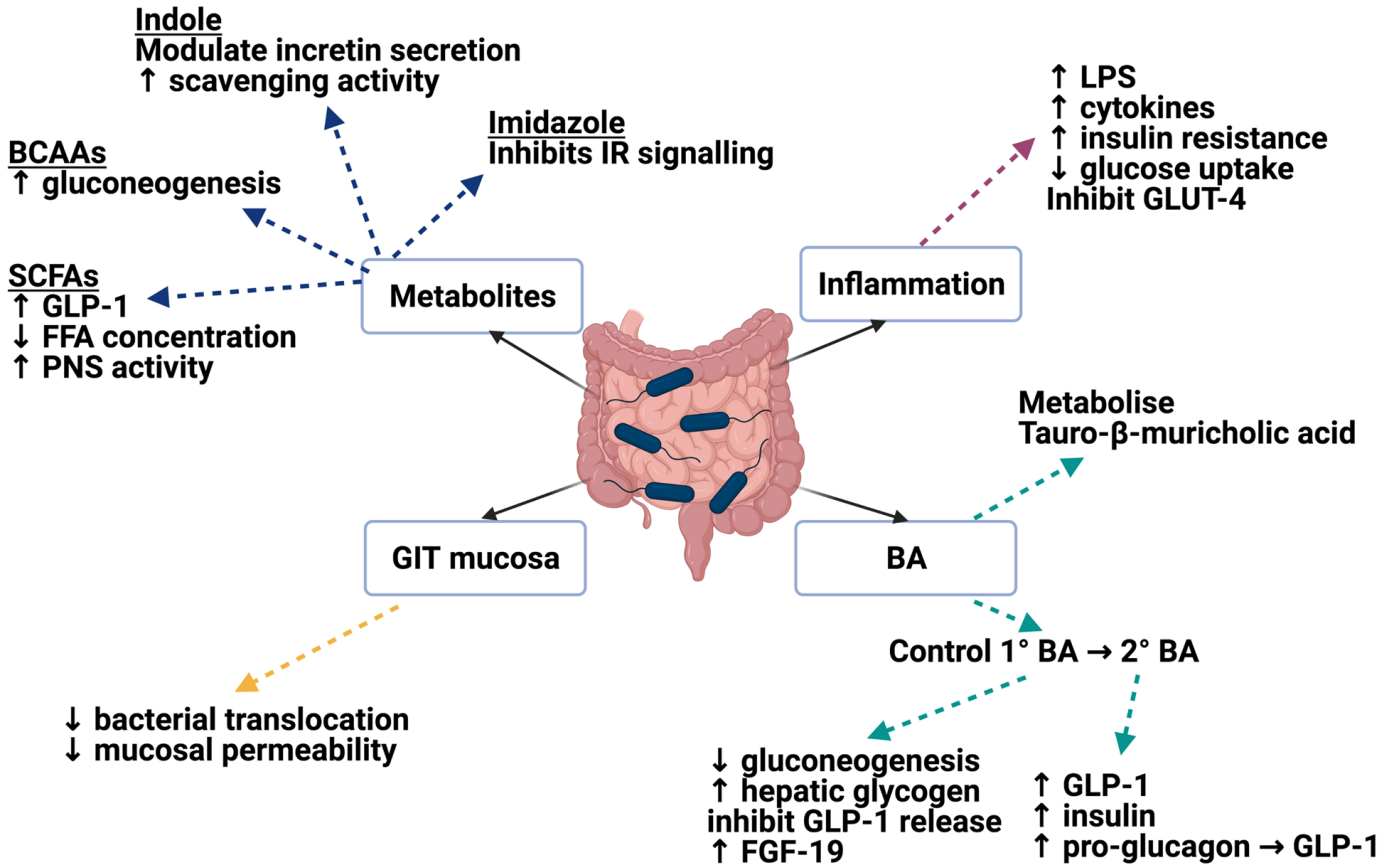
Microbiota influence on glucose homeostasis

Gut microbiota have the ability to alter host glucose homeostasis through multiple mechanisms including: the production of metabolites during fermentation and their resulting secondary effects; activation of inflammatory cascades leading to the release of cytokines; disrupting the permeability of the intestinal mucosal barrier allowing the influx of toxins; and direct signalling action through incretin secretion. These mechanisms have been discussed in great detail elsewhere, but we will summarise the main influencing factors below [68]. T2DM patients demonstrate an enrichment in their membrane transport of sugars, branched chain amino acids (BCAA) transportation, methane metabolism, xenobiotic degradation and metabolism, and sulphate reduction. The same cohort displayed reduced levels of bacterial chemotaxis, flagellar assembly, butyrate biosynthesis and metabolism of cofactors and vitamins [18]. Figure 1 provides a diagrammatic summary.

## Gut microbiota metabolites

SCFAs, BCAAs, succinate, indole and imidazole are all microbial metabolites produced during anaerobic fermentation in the gut and act as central components in microbe-to-host signalling pathways [69, 70]. These metabolites are predominantly produced from microbiota genera such as *Akkermansia*, *Prevotella*, *Ruminococus*, *Coprococcus*, *Faecalibacterium*, *Eubacterium*, *Roseburia*, *Clostridium*, *Bacteroides*, *Lactobacillus*, *Streptococcus*, *Propionibacterium* and *Fusobacterium* [71–73]. As discussed earlier, the majority of these particular microbiota are depleted in patients with T2DM.

Butyrate, acetate and propionate are the most abundant SCFAs produced by intestinal fermentation of dietary fibre [74, 75]. Acetate and propionate are mostly produced by the phylum Bacteroidetes, while butyrate is produced by the Firmicutes [76]. SCFAs are directly utilised as an energy source by the intestinal mucosal cells or transferred to the systemic circulation to generate an important source of energy for the host and have the ability to behave as signaling molecules [74].

SCFAs strongly influence glucose metabolism through the coupling action with selected G-protein-coupled receptors (GPRs). These are predominantly expressed in adipose tissue, the intestine, and immune cells. GPR43 and GPR119 stimulation promotes the secretion of the incretin GLP-1 from enteroendocrine L-cells [77–79]. GLP-1 intensifies glucose-induced insulin release from β-cells, suppresses glucagon secretion, protects β-cells from apoptosis, promotes β-cell proliferation and prolongs intestinal transit time [80].

Stimulation of the receptor GPR41 by butyrate and propionate has the ability to induce intestinal gluconeogenesis through two different mechanisms of action. Firstly, by acting as a GPR41 agonist which enhances intestinal gluconeogenesis gene expression and secondly through a gut–brain neural circuit involving GPR41 [81].

SCFAs can also directly impact hepatic glucose metabolism, decreasing glycolysis and gluconeogenesis, increasing glycogen synthesis and lowering plasma fatty acid concentration [29, 82]. SCFAs have the ability to activate parasympathetic activity subsequently increasing appetite and promoting glucose-stimulated insulin secretion [83].

SCFAs have been demonstrated to enhance glucose uptake peripherally, by increasing the expression of glucose transporter type 4 (GLUT4), through the action of AMP-activated protein kinase (AMPK) activity. Secondly, in skeletal muscle, SCFAs have the capability of reducing glycolysis resulting in secondary accumulation of glucose-6-phosphate leading to greater glycogen synthesis [84, 85].

Acetate, the most abundant SCFA, is taken up by the intestinal epithelium, transported to the liver via the portal vein, and eventually distributed to peripheral tissues where it is metabolised [86]. Systemic acetate has the capability of crossing the blood–brain barrier where it can activate acetyl-CoA carboxylase leading to the enhancement of the expression of neuropeptides which induces hypothalamic neuronal activation and suppresses appetite [87].

Butyrate is the principal substrate and energy source for colonocytes providing at least 60–70% of colonic mucosa energy requirements, essential for their proliferation and differentiation [29, 30]. Butyrate is important in maintaining colonic epithelium homeostasis mainly by utilising its anti-inflammatory properties, thereby preventing the production of reactive oxygen and nitrogen species generated in oxidative stress [31, 88]. Sanna et al. reported that the abundance of butyrate-producing microbiota is associated with an improved insulin response during an oral glucose tolerance test (an indication of improved β-cell function) [89].

Intestinally produced propionate is a known preferred precursor for gluconeogenesis of which approximately 50% is utilised in this manner [69, 90]. Propionate enters the tricarboxylic acid (TCA) cycle and is converted into succinyl-CoA via three successive reactions. The resulting succinyl-CoA re-enters the TCA cycle and is converted into oxaloacetate, the gluconeogenesis precursor [91].

Increased intestinal propionate delivery has been associated with enhanced β-cell function and glucose-stimulated insulin secretion that was independent of alterations in GLP-1 levels. Propionate also provided protection for the human islets through the direct inhibition of apoptosis induced by inflammatory cytokines [92]. Lastly, supplementing overweight patients with intestinal propionate resulted in reduced energy intake and adiposity, and intensified plasma levels of peptide YY (PYY) and GLP-1 [93].

It is possible to improve T2DM control through the introduction of a high-fibre diet. Encouraging patients to ingest a high-fibre diet has been demonstrated to improve the quantities of SCFA-producing microbiota, leading to a reduction in HbA1c levels facilitated by an increase in the production of GLP-1 [94]. Patients receiving a high-fibre diet had greater reductions in HbA1c levels and a higher proportion of this cohort achieved adequate glycaemic control (HbA1c < 7%) compared with the control group [94]. A further clinical study [95], advising the ingestion of a Mediterranean diet (rich in fibre), has also reported improvements in glucose and insulin sensitivity in individuals with high cardiometabolic risk. Greater postprandial plasma butyrate concentrations were found associated with increases in the abundance of the species *Intestinimonas butyriciproducens* and *Akkermansia muciniphila*. Of note, the butyrate concentrations directly correlated with postprandial insulin sensitivity, evaluated by the oral glucose insulin sensitivity (OGIS) model [95].

The bacterial fermentation of dietary fibre produces large amounts of succinate that improves glycaemic control through the activation of intestinal gluconeogenesis [96], which is also true of the SCFAs butyrate and propionate [81, 97]. Increases in a small number of essential amino acids including the BCAAs and the aromatic amino acids have been reported to be associated with a five-fold increased risk of developing T2DM in the future [98]. Raised plasma levels of BCAAs has also been demonstrated to be characteristic of insulin resistance, correlating with two specific bacterial species, *Prevotella copri* and *Bacteroides vulgatus* [31]. Insulin-resistant patients display enriched BCAA biosynthesis and are found to be deprived of genes encoding bacterial inward transporters for these particular amino acids [31]. In rodents, it was demonstrated that the species *Prevotella copri* can induce insulin resistance, exacerbate glucose intolerance and intensify BCAA levels [31].

Indolepropionic acid, a metabolite generated by bacterial aromatic amino acid catabolism is highly correlated with dietary fibre intake and appears to reduce the risk of developing T2DM. It provides potent radical scavenging activity raising the suggestion that it may provide protection for the pancreatic β-cell from damage associated with metabolic and oxidative stress [32]. It may also be involved in the modulation of incretin secretion from enteroendocrine L-cells by inhibiting voltage-gated potassium channels, triggering GLP-1 secretion [99, 100].

Imidazole propionate, produced from the degradation of histidine by gut microbiota, impairs the ability of cells to correctly respond to insulin by acting as an inhibitor of the intracellular insulin receptor signalling cascade [101].

## Bile acids

BAs are steroid carboxylic acids derived primarily from cholesterol through the action of the rate‐limiting enzyme 7α‐hydroxylase (CYP7A1), which are then conjugated to glycine or taurine before being secreted in bile. Greater than 95% are reabsorbed in the terminal ileum and colon through the enterohepatic circulation [102, 103]. The main function of BAs is the digestion and absorption of lipids and lipid-soluble vitamins within the small intestine. *Lactobacillus*, *Bifidobacterium*, *Enterobacter*, *Bacteroides* and *Clostridium* are the main gut microbiota that influence BA synthesis, modification and signalling. They have the ability to control the conversion of primary BAs (cholic acid and chenodeoxycholic acid) into secondary BAs (deoxycholic and lithocholic acids), through the process of deconjugation and the ability to metabolise the naturally occurring FXR antagonist tauro-β-muricholic acid [102–105]. In turn, BAs contribute towards intestinal homeostasis by suppressing bacterial colonisation and growth in the intestine because of their strong antimicrobial activity [106]. In addition to roles in intestinal digestion and absorption, BAs have the ability to exert important metabolic effects acting as hormones.

BAs can adjust glucose metabolism through receptor coupling signalling using both the FXR and G-protein receptor 5 (TGR-5) [107]. FXR coupling is only possible by primary BAs and has the ability to reduce gluconeogenesis, promote hepatic glycogen production, inhibit the release of GLP-1 and stimulate the secretion of fibroblast growth factor (FGF-19) from the ileum. FXR signaling inhibits the expression of gluconeogenic genes, such as those encoding phosphoenolpyruvate carboxykinase, fructose-1, 6-biphosphatase-1, and glucose-6-phosphatase [108]. FGF-19 regulates BA synthesis by reducing the expression of CYP7A1, inhibiting glucose production and inducing glycogen synthesis. TGR-5 (bound only by secondary BAs) coupling results in GLP-1 secretion from intestinal L-cells, increases glucose-stimulated insulin release and promotes the conversion of pro-glucagon to GLP-1. In skeletal muscles and brown adipose tissue, BA-TGR5 signaling encourages thyroxine (T4) conversion to the biologically active triiodothyronine (T3) through the stimulation of type 2 iodothyronine deiodinase, resulting in greater energy expenditure. Coupling of both receptors encourages the production of insulin from pancreatic β-cells [102, 109–111].

Evidence suggests that manipulation of the BA pool using BA sequestrants improves glycaemic control in patients with T2DM. BA sequestrants bind BAs in the intestine to form a nonabsorbable complex resulting in interruption of the enterohepatic circulation. The mechanisms underlying the blood glucose-lowering effect of BA sequestrants are poorly understood, but are believed to involve the disruption of the BA pool composition, enhancing hepatic glucose metabolism, increasing the release of incretin hormones and inducing alterations in gut microbiota composition [112, 113].

## Gastrointestinal barrier function

The intestinal mucosal lining acts as a preventative barrier to undesirable interactions with potentially harmful substances and plays an integral role in the regulation of the immune system [114]. T2DM is well understood to have significantly enhanced permeability in the gut allowing for the translocation of bacteria across the gut epithelium resulting in host metabolic endotoxaemia triggering low-grade inflammation. The resulting effects can initiate β-cell destruction and insulin resistance [50, 115]. As already described, the genera *Faecalibacterium*, *Roseburia* and *Bifidobacterium* are all recognised in their abilities to provide protection against bacterial translocation and reduce intestinal permeability [116, 117]. T2DM patients are known to have depleted abundances of these particular microbiota.

## Inflammatory response

T2DM is characterised by a state of chronic low-grade inflammation combined with abnormal expression and production of numerous inflammatory mediators [118–120]. Individuals with T2DM have reduced amounts of butyrate-producing microbiota, encouraging low-grade inflammation in the gut [21, 121]. Gut microbiota activate host inflammation and insulin resistance through the activity of lipopolysaccharide (LPS), an essential component of Gram-negative bacteria cell walls [35, 122, 123]. Bacterial fragments and LPS are recognised by innate toll-like receptors (TLRs), particularly TLR-4, triggering the activation of the intracellular signalling pathway NF-κB and the release of pro-inflammatory cytokines [123–125]. LPS release also stimulates localised immune responses through high-affinity coupling with the NLRP3 inflammasome and NOD-like receptors (NLRs) expressed on macrophages and dendritic cells [126]. The activation of serum kinases (Jnk and IKK) in the inflammatory NF-κB cascade induces phosphorylation of the insulin receptor substrate (IRS) serine, worsening insulin resistance [52].

The release of pro-inflammatory cytokines disrupts glucose metabolism and insulin signalling. T2DM patients display elevated levels of TNF-α, which is strongly associated with altered glucose tolerance, enhanced insulin resistance and islet dysfunction [127–129]. TNF-α has the capability to up-regulate the transcription of suppressor of cytokine signaling-3 (SOCS-3) which couples to tyrosine-960 of the insulin receptor preventing IRS-1 binding to the insulin receptor (IR). This then leads to the degradation of IRS-1 and the disruption of the insulin signalling pathway [130, 131].

Interleukin-1 (IL-1), an inflammatory cytokine of the interleukin family has the potential to reduce the expression of IRS-1, inhibit the translocation of the GLUT-4 to the plasma membrane and reduce insulin-stimulated glucose uptake [132]. Recent work has illustrated that an IL-1 receptor antagonist (IL-1RA) and IL-1β-specific antibody treatment improved glucose metabolism and insulin secretion in T2DM patients [133, 134].

IL-6 has been identified as an independent predictor of T2DM [135]. It exerts long-term inhibition on gene transcription of IRS-1, GLUT4, and peroxisome proliferator-activated receptors (PPARs), as well as significantly reducing insulin-stimulated tyrosine phosphorylation and insulin-stimulated glucose transport [136].

## Prebiotics, probiotics and synbiotics

Prebiotics, probiotics and synbiotics are attractive dietary adjuncts with the capability of manipulating the intestinal microbiota composition with the aim of creating an environment for the improvement in glucose metabolism. A growing literature base supports the clinical usage of the addition of prebiotics, probiotics and synbiotics for improving glycaemic control in patients diagnosed with T2DM [137, 138]. It is challenging however, due to the heterogeneity between study methodology (study duration, quantity of supplement, patient demographics) which hinders study comparison and data remains limited by the poor availability of studies, relative small size of individual studies and the clear lack of microbiota data.

Probiotics are living microorganisms that, when administered in adequate quantities, confer benefits to an individual’s health [139] whereas prebiotics are food components such as indigestible polysaccharides or fibre that beneficially affect the host by selectively stimulating the growth and/or activity of one or a limited number of intestinal microbiota [140]. Lastly, ‘a mixture comprising live microorganisms and substrate selectively utilised by host intestinal microbiota to confer ‘a host health benefit’ is described as a synbiotic [141].

Evidence suggests that probiotics are able to improve the intestinal microbiota community leading to greater T2DM control with associated enhanced intestinal integrity, decreased circulating LPS, decreased endoplasmic reticulum stress and improved peripheral insulin sensitivity [142]. Tao et al., performed a meta-analysis focusing on the effects of probiotic supplementation on HbA1c levels, fasting blood glucose (FBG) and insulin resistance in T2DM patients. A total of 15 randomised control trials (RCTs) involving 902 patients were included. The results showed that probiotics may reduce HbA1c levels (*p* = 0.02), FBG (p = 0.003), and insulin resistance (*p* < 0.00001) from baseline [143]. As mentioned earlier, limited studies comment on microbiota alterations. Two studies mentioned microbiota analysis following probiotic addition and reported changes in bacterial composition. Andreasen et al. [144]., reported a significant enhancement in the abundance of the species *Lactobacillus acidophilus* from near non-detectable levels pre-intervention. Firouzi et al. [145], reported significant increases in the quantities of the genus *Bifidobacterium* (4.5 fold) and *Lactobacillus* species (twofold).

Prebiotic supplementation is associated with improved glycaemic control however as per with probiotic study reporting, heterogeneity in methodology is also vast resulting in inconclusive literature. Wang et al. [146]., published the most comprehensive meta-analysis to date, which included 33 RCTs involving 1346 participants spread across healthy, obese and T2DM cohorts. Focusing solely on the prediabetic and T2DM cohorts, compared with the control, the relative reduction of the FBG, HbA1c levels, fasting insulin concentration and insulin sensitivity was 7.15, 7.00, 16.58, and 25.34% of their baseline values after supplementation [146]. A daily supplement dose greater than 10 g and for a minimum duration of 42 days was recommended for consistent improvement across the glycaemic indicators.

It is unclear whether the observed effects are related to gut microbiota modification or because of the greater availability of substrate for fermentation. There is a sustained lack of microbiota analysis across the literature directly attributed to the improvements in glucose levels. Birkeland et al. [147], demonstrated that six weeks addition of a prebiotic can produce a significant bifidogenic effect and improve faecal SCFA concentrations however no effect was seen on overall microbial diversity. Secondly, Pedersen et al. [148], showed that prebiotic supplementation can increase bacterial diversity, as assessed by the Shannon and inverse Simpson indices, and richness in T2DM patients. However, no statistical improvements in glucose control were observed after twelve weeks of dietary treatment.

Lastly, Sheth et al. [149]., introduced a synbiotic to sixty pre-hypertensive patients with T2DM (two species of *Lactobacillus* and *Bifidobacterium* each, one species of *Streptococcus* and yeast, and 300 mg oligosaccharide). Increases in both the genera *Lactobacillus* (32.6%) and *Bifidobacterium* (131.6%) and a significant reduction in enteric pathogens (44.6%) were reported following the intervention along with improvements in fasting blood glucose (3.3%) and HbA1c levels (14%).

There is increasing evidence that the addition of prebiotics, probiotics and synbiotics can improve glycaemic control. Detailed work is required in designing robust methodology to identify whether these positive changes are directly attributable to the modification of the intestinal microbiota and the complex metabolic mechanisms involved. Once this relationship is better understood, the potential to utilise these dietary additions in the management of T2DM can begin.

## Faecal microbiota transplantation

Faecal microbiota transplantation (FMT) is the transfer of minimally manipulated pre-screened donor stool, into the GIT of an identified ‘diseased’ patient with the aim of correcting the dysbiotic state, increasing overall diversity and restore the functionality of the microbiota [150]. Currently, FMT is only recommended for the treatment of recurrent *Clostridium difficile* infection (rCDI) with resolution rates exceeding 89% [151]. There are a significant number of ongoing studies exploring other potential indications including T2DM. FMT is believed to have better potential than dietary supplements such as probiotics because FMT has the capability of transferring entire donor microbiota communities, including their metabolites, with the perceived enhanced capability to correct microbiota disruption over single microbial targets [152].

There is currently limited evidence for the use of FMT in T2DM but human studies investigating the clinical effects of FMT in patients with metabolic syndrome can be used to stimulate interest. Vrieze et al. [153], reported the improved insulin sensitivity of male recipients diagnosed with metabolic syndrome and the enhanced abundance of butyrate-producing intestinal microbiota (the species *Roseburia intestinalis*), six weeks after receiving allogenic microbiota. Secondly, a study reported that patients with metabolic syndrome, observed that HbA1c levels were significantly reduced after allogenic FMT, and was associated with changes in intestinal microbiota composition [154]. Reduced gene richness in participant baseline microbiota before allogenic FMT was related with improved clinical outcome. It should be mentioned that the clinical benefits in both studies deteriorated with time and there was considerable individual variability.

Further detailed work is required in order to explore the potential of FMT, particularly in metabolic disease (T2DM) such as identifying optimal donor microbiome characteristics; calculating appropriate dosing frequency and thresholds in the need for replenishing treatments in order to achieve longevity of microbiota engraftment; whether there is a requirement for recipient preparation; and lastly whether recipient host factors have the ability to modulate treatment efficacy. It is possible that manipulating the gut microbiota using techniques such as FMT might be an extremely promising therapeutic option in the management of T2DM.

## Conclusion: future direction

T2DM is a complex multi-system disorder which can have life changing complications if not identified and treated appropriately. As described in this article, particular gut microbiota may contribute heavily to this development through the alteration of glucose metabolism pathways. As the T2DM population grows, this enables access to a vast number of patients for investigation. However, more meticulous work is required to disentangle a ‘common’ microbiota profile, given that these patients tend to be on multiple prescribed medications and have other related/unrelated comorbidities. This microbiota profile may be an individual or a collective group of gut microbiota but conclusive evidence is needed in order to assist the identification of the ‘at-risk’ population before the onset of disease.

Once a common profile is well understood, it will enable the exponential growth of microbiota targeted therapeutics in order to establish a strong evidence base on the metabolic effects of altering host microbiota. Treatment regimes that need to be thoroughly investigated include the use of prebiotics, probiotics and facilitated microbiota transfer (FMT), with the end goal of simplified early intervention in identified at-risk populations. This would reduce unnecessary secondary health complications with significant cost savings to the health service.

## Data Availability

Data sharing not applicable to this article as no datasets were generated or analysed during the completion of this review.

## Abbreviations

AMPK: AMP-activated protein kinase
BA: Bile acid
BCAA: Branched chain amino acid
BMI: Body mass index
CYP7A1: Cholesterol 7 alpha‐hydroxylase
FBG: Fasting blood glucose
FGF-19: Fibroblast growth factor
FMT: Faecal microbiota transplant
FXR: Farnesoid X receptor
GIT: Gastrointestinal tract
GLUT-4: Glucose transporter type 4
GLP-1: Glucagon-like peptide 1
GPR: G-protein receptor
GUDCA: Glycoursodexoycholic acid
HbA1c: Glycated haemoglobin
HFD: High fat diet
IA: Islet autoimmunity
IKK: Inhibitor of nuclear factor-κB kinase complex
IL-1: Interleukin 1
IL-1RA: Interleukin-1 receptor antagonist
IR: Insulin receptor
IRS: Insulin receptor substrate
JNK: C-Jun N-terminal kinase pathway
LPS: Lipopolysaccharide
NF-κB: Nuclear factor kappa light chain enhancer of activated B-cells
NLRs: NOD like receptors
NLRP3: Nucleotide-binding domain-like receptor protein 3
OGIS: Oral glucose insulin sensitivity
OGTT: Oral glucose tolerance test
PPARs: Peroxisome proliferator-activated receptors
PYY: Peptide tyrosine tyrosine
rCDI: Recurrent *Clostridium difficile* infection
RCT: Randomised clinical trial
SCFA: Short chain fatty acid
SOCS-3: Suppressor cytokine-3
T3: Triiodothyronine
T4: Thyroxine
TCA: Tricarboxylic acid
TEDDY: The Environmental Determinants of Diabetes in the Young study
TGR-5: G-protein receptor 5
TLRs: Toll-like receptors
T1DM: Type one diabetes mellitus
T2DM: Type two diabetes mellitus
TNF-α: Tumour necrosis factor alpha
UK: United Kingdom
UN: United Nations
WHO: World Health Organisation
